# Sequential Evaporation for Scalable Hybrid Processing of Perovskite/Silicon Tandem Solar Cells

**DOI:** 10.1021/acsami.6c07582

**Published:** 2026-06-12

**Authors:** Lorenzo Mardegan, Mingjie He, Badri Vishal, Thomas Allen, Anand Subbiah, Arsalan Razzaq, Adi Prasetio, Anil R. Pininti, Martin Bivour, Juliane Borchert, Ahmed Ali Said, Stefaan De Wolf

**Affiliations:** † Center for Renewable Energy and Storage Technologies (CREST), Physical Sciences and Engineering, Division (PSE), King Abdullah University of Science and Technology (KAUST), Thuwal 23955-6900, Saudi Arabia; ‡ 28477 Fraunhofer Institute for Solar Energy Systems ISE, Heidenhofstr. 2, 79110 Freiburg, Germany; § Chair for Photovoltaic Energy Conversion, Department of Sustainable Systems Engineering (INATECH), University of Freiburg, 79110 Freiburg, Germany

**Keywords:** sequential thermal evaporation, hybrid vacuum-solution processing, sequentially deposited inorganic scaffold, perovskite solar cells, perovskite/silicon tandem solar cells, industry-compatible processing

## Abstract

The conformal coating of perovskites on textured silicon for tandem solar cells requires scalable deposition methods, for which hybrid vacuum-solution processing, using an evaporated inorganic scaffold (Pb/Cs halides) followed by solution conversion, is promising. Yet multisource coevaporation of the scaffold, as commonly used for lab-scale devices, is complex and costly to implement at an industrial scale. Here, we investigate sequential single-source evaporation of the inorganic scaffold as an industrially viable alternative. Five sequentially evaporated scaffold stacks were compared to a coevaporated reference. Despite differences in PbI_2_ conversion, halide distribution, and morphology, all showed similar single-junction device power conversion efficiencies (∼19%). The best scaffold (a PbI_2_/CsBr/PbI_2_/CsBr/PbI_2_ stack) achieved 28.4% in perovskite/silicon tandems without molecular additives, matching the coevaporated reference (28.3%). This work demonstrates that sequential deposition of the inorganic scaffold offers a scalable route to high-efficiency tandems.

## Graphical abstract

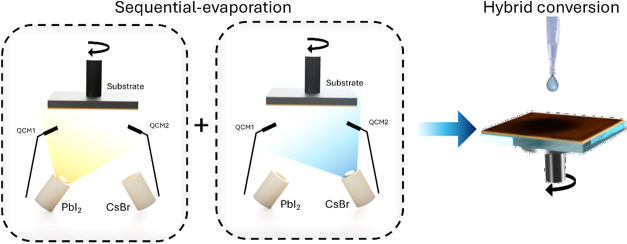

## Introduction

Perovskite solar cells (PSCs) have undergone rapid improvements in power conversion efficiency (PCE) over the past decade, now approaching 27% at the single-junction level.^1^ Perovskite absorbers are also well suited for integration with Si bottom cells in tandem configurations, where monolithic perovskite/silicon tandem solar cells have already surpassed the theoretical single-junction PCE limit, achieving record values above 34% and demonstrating significant promise for next-generation high-efficiency photovoltaic modules.^2,3^

The scalability of perovskite thin-film processing, from laboratory research to industrial production, remains a widely investigated topic in the literature, largely due to the diversity of available fabrication methods, each presenting distinct advantages and limitations. In the case of perovskite/silicon tandems, an additional challenge arises from the micron-scale pyramidal texture and residual saw-damage roughness of the silicon bottom cell. Within this context, the hybrid deposition approach, combining vacuum deposition of an inorganic scaffold with its subsequent conversion into the perovskite phase via solution processing (Figure 1), emerges as a particularly promising strategy. This method offers enhanced thickness control, improved film uniformity, and excellent conformality over complex substrate geometries. In perovskites/silicon tandems, this approach has led to certified PCEs above 32%.^4^ Moreover, this approach has also already been successfully demonstrated in large-area solar cells and mini-modules, achieving PCEs of 26.6% (certified) and 27.1% for tandems with aperture areas of 64.64 cm^2^ and 110 cm^2^, respectively, and 21.16% (certified) for a perovskite single-junction module of 810 cm^2^.^5–7^

**Figure 1:**
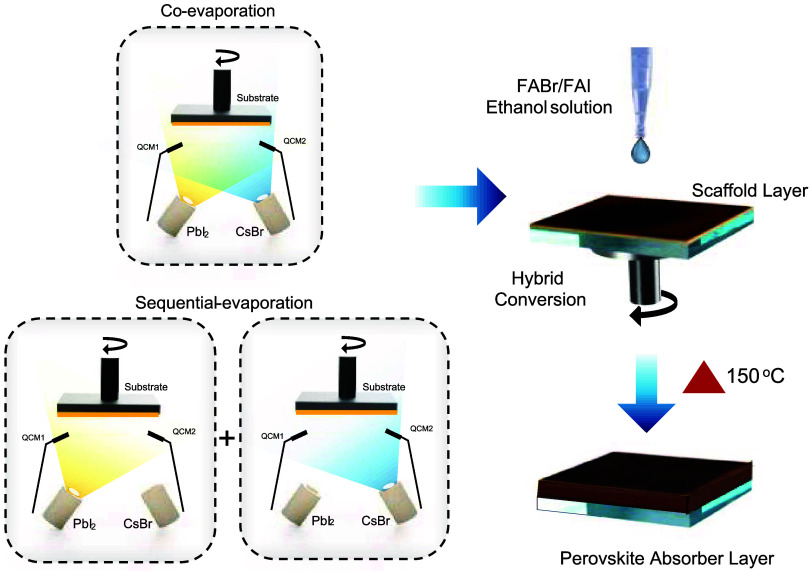
Illustration of the vacuum-solution hybrid method, highlighting the coevaporation vs sequential evaporation step for the deposition of the PbI_2_–CsBr inorganic scaffold stacks, followed by spin-coating of the formamidinium halide (FAX) solution and annealing under controlled humidity to obtain the perovskite α-phase.

Although well established at the laboratory scale, several adaptations are required to the hybrid approach to enable cost-efficient large-scale production, especially in terms of materials consumption and throughput. Conveniently, the solution-based conversion step of the hybrid method is compatible with scalable printing techniques such as slot-die coating, inkjet printing, blade coating and spray coating.^5–12^ However, for the inorganic scaffold, most results to date have focused on multisource coevaporation, relying on laboratory-scale thermal evaporators. These systems are typically equipped with multiple point sources positioned tens of centimeters from the substrate to enable homogeneous coevaporated films.

Industrial evaporators, however, are designed for high-throughput, large-area coating applications. In these systems, improving material utilization efficiency, defined as the fraction of evaporated material that is ultimately deposited onto the active substrate area, is a key consideration. To achieve this, the source-to-substrate distance is typically minimized, and linear evaporation sources are employed, as they are well suited for high-throughput in-line processing.^13^ However, in this layout, coevaporation with overlapping deposition zones and rate monitoring are technically more challenging. In this context, single-source sequential depositions could simplify the vacuum chamber design and rate monitoring setup, reduced to measuring single layer thicknesses (*i.e*, by ellipsometry, UV–vis spectroscopy, X-ray diffraction) rather than a real-time feedback loop for multisource rate control.^14–18^

Replacing conventional coevaporation of the inorganic scaffold in the evaporation/solution hybrid method with sequential evaporation offers a potential pathway toward true high-throughput processing while simplifying system complexity. Sequential deposition also permits higher PbI_2_ deposition rates, which are beneficial for optimal scaffold conversion and throughput.^19^ Simulations based on laboratory-scale point-source rates suggest that depositing a ∼ 300 nm scaffold at high throughput (equivalent to ∼1000 wafers per hour) would require three linear sources, one for CsX and two for PbI_2_.^20^

Despite these obvious advantages at the industrial scale, the sequential deposition of the inorganic scaffold has rarely been applied in the context of the hybrid method.^9,10^ Very recently, a study demonstrated that sequential deposition of CsCl and PbI_2_ using a vacuum-solution hybrid approach enables 1.70 eV perovskites with PCEs exceeding 20%.^21^ By comparing coevaporated scaffolds with CsCl/PbI_2_ bilayer stacks, the authors showed that both the scaffold and the resulting perovskite’s morphology, microstructure, and elemental distribution are strongly dependent on the chosen deposition method. In these stacked CsCl/PbI_2_ configurations, uniform Cs^+^ incorporation is a key determinant of high-quality films, with the high diffusivity of Cs^+^ promoting homogeneous distribution throughout the final perovskite.

In this complementary study, we systematically compare the conventional coevaporation of PbI_2_ and CsBr (hereafter denoted as Pb and Cs, respectively) with five distinct single-source sequential deposition schemes: Pb/Cs, Cs/Pb/Cs, Pb/Cs/Pb, Cs/Pb/Cs/Pb, and Pb/Cs/Pb/Cs/Pb. Using a suite of physical characterization techniques, we reveal pronounced differences in the resulting perovskite bandgap, residual PbI_2_ content, and halide distribution, depending on the number and sequence of PbI_2_ and CsBr layers. Despite these substantial scaffold variations, device performance remains largely consistent, with champion power conversion efficiencies (PCEs) of ∼ 20% for small-area cells (0.1 cm^2^).

Finally, both the standard coevaporated scaffold and the Pb/Cs/Pb/Cs/Pb sequential scaffold were integrated into additive-free perovskite/silicon tandem solar cells to assess performance on textured surfaces. Remarkably, both scaffolds achieved comparable PCEs, with the sequentially evaporated scaffold exceeding 28% (1.04 cm^2^) and outperforming the coevaporated control, highlighting the robustness and scalability of the sequential deposition approach.

## Results and Discussion

To assess the viability of sequential PbI_2_ and CsBr deposition to form the inorganic scaffold in hybrid processing at an industrial scale, we conducted in-depth characterization of the perovskite layers resulting from five different scaffold stacks (summarized in Figure 2a) and compared them to a coevaporated scaffold reference. The most commonly reported Pb:Cs evaporation rate ratio for hybrid-processed perovskites is 1:0.1.^4,22,23^ To ensure identical stoichiometric composition across all stacks before conversion, the relative thicknesses of PbI_2_ and CsBr were derived from the process parameters (rate ratio) and the final thickness of the coevaporated scaffold (see Experimental Methods).

**Figure 2:**
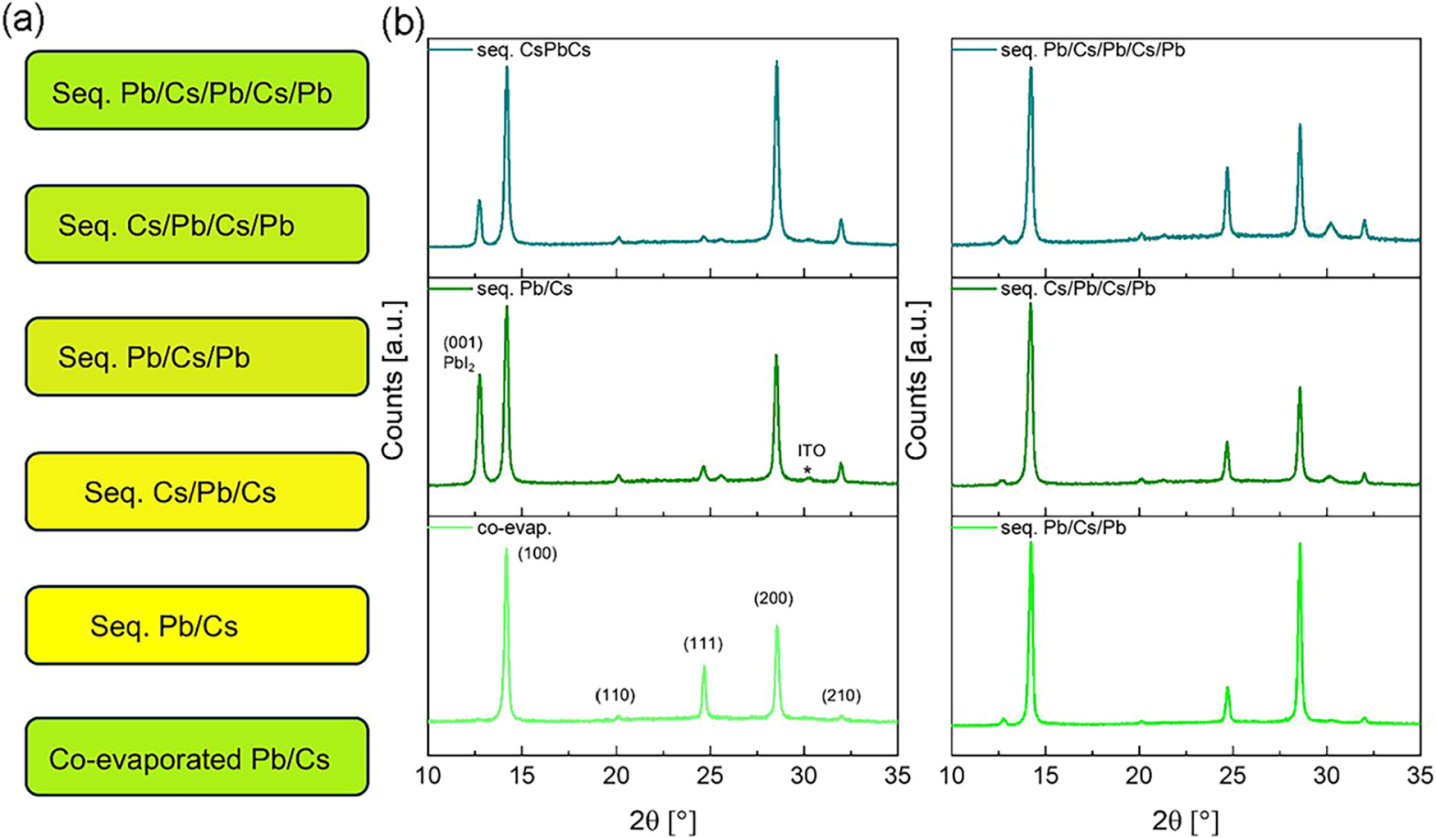
(a) Schematic of the inorganic scaffolds, prepared by coevaporation and sequential evaporation, tested in this work. (b) Perovskite XRD patterns converted from each sequentially deposited scaffold.

A key factor when comparing coevaporated and sequentially evaporated processes is the elemental distribution. As previously noted, the deposition route significantly influences the distribution of inorganic components in the final perovskite.^21^ We therefore first characterized the perovskite films using XRD. Although XRD does not directly probe elemental homogeneity, it provides valuable insight into the extent of PbI_2_ conversion, specifically, whether the PbI_2_ scaffold has fully reacted to form the perovskite phase.

The XRD patterns shown in Figure 2b reveal that the five scaffolds convert to perovskite with varying amounts of residual PbI_2_. The coevaporated scaffold yields a standard diffraction pattern, characterized by a strong orientation toward the (100) direction and no detectable residual PbI_2_ throughout the film. It is worth noting here that the spin-coating step also plays a critical role, and even with optimized FA^+^ concentration and skilled handling, the resulting perovskite films can exhibit significant batch-to-batch variation in residual PbI_2_ content (Figure S1). Here, it is worth noting that the role of residual PbI_2_ has been extensively investigated,^24,25^ and correlated to improved device performance in solution-processed perovskite solar cells (MAPbI_3_-based), mainly due to templating and passivation effects,^26–29^ with also important concerns regarding long-term stability.^30–32^ At the same time, for hybrid perovskites, the role of residual PbI_2_ remains uncertain. First, the perovskite conversion mechanism fundamentally differs from that of fully solution-processed systems, and possible PbI_2_ templating effects must therefore be addressed. Second, although complete PbI_2_ conversion is generally considered desirable, particularly for tandem devices,^22^ based on our experience, we have observed both high- and low-performing single-junction devices containing a significant excess of residual PbI_2_. Therefore, we hypothesize that the effects of residual PbI_2_ in the context of the hybrid conversion are highly dependent on its spatial location within the film (buried or top interface, grain boundaries), which in turn might depend on the organic solution composition and molecular additives.

On the other hand, the observed batch-to-batch variations might arise from small thickness differences originating from the vacuum deposition process, for example due to QCM crystal lifetime, as well as from variations in solution concentration.

Turning to the perovskites obtained from the five sequential scaffold stacks, a clear trend emerges. As shown in Figure 2b, the perovskite derived from the Pb/Cs stack exhibits a substantial amount of residual PbI_2_, identified by the characteristic (100) peak at 12.7°. Interestingly, as the number of PbI_2_ and CsBr layers increases, the intensity of the PbI_2_ reflection diminishes. This suggests that stacks composed of multiple thinner layers are easier to homogenize during conversion compared to stacks consisting of only two thicker layers, as for the Pb/Cs sample.

Another factor that appears to influence the final perovskite film is the deposition order of the scaffold layers. The perovskite obtained from the Cs/Pb/Cs stack exhibits significantly more residual PbI_2_ than that converted from Pb/Cs/Pb. These results further support the notion that scaffold stacks composed of thinner PbI_2_ and CsBr layers, as in Pb/Cs/Pb/Cs/Pb, promote an improved PbI_2_ utilization during the conversion step.

Interestingly, the relative elemental distributions extracted from TEM analysis (Figure S2) and shown in Figure 3a–c reveal distinct profiles for perovskites derived from coevaporated, Pb/Cs, and Pb/Cs/Pb/Cs/Pb scaffolds.

**Figure 3:**
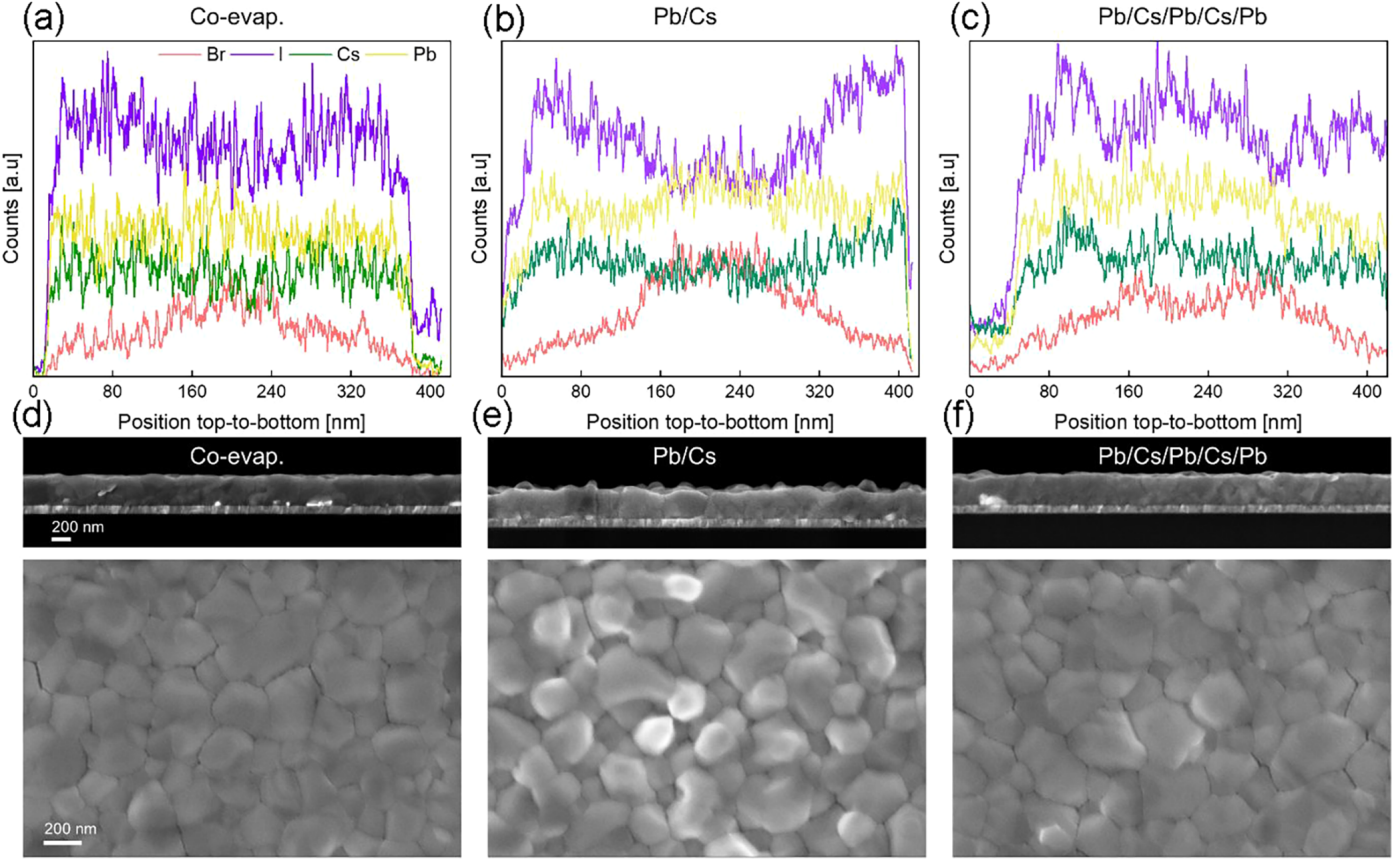
(a–c) Cross-sectional elemental distribution extracted from HAADF-STEM-EDS (Supporting Figure 2) of the converted perovskite phase obtained from coevaporated, Pb/Cs, and Pb/Cs/Pb/Cs/Pb scaffolds, respectively. (d–f) SEM cross-sectional and surface images of the converted perovskite phase, obtained from coevaporated, Pb/Cs, and Pb/Cs/Pb/Cs/Pb scaffolds, respectively.

Here, it is worth mentioning that HAADF-STEM-EDS measurements detect the elements, regardless of their chemical state (e.g., Pb^0^ vs Pb^2+^), therefore, in this context, we will adopt the following notation: Pb, Cs, I, and Br. Contrary to expectations, Cs (green line) is distributed homogeneously across the film thickness in all samples, with only a slight depletion around the midpoint of the Pb/Cs film (Figure 3b). Regarding Pb (yellow line), a constant distribution is observed throughout the coevaporated and Pb/Cs/Pb/Cs/Pb samples. In contrast, the Pb/Cs perovskite exhibits a rise in Pb that coincides with the region of Cs depletion noted above.

The halide trends reveal overlapping I depletion and Br enrichment (orange and purple lines), a feature common to all samples. Specifically, in both the coevaporated and Pb/Cs samples, Br increases toward the middle of the film. In the Pb/Cs/Pb/Cs/Pb sample, however, two smaller Br increments appear near the top and bottom interfaces, likely corresponding to the Pb/Cs interfaces within the scaffold. At the same time, I follows a complementary behavior, increasing where Br is lower. The combined XRD and TEM data point to a complex scenario in which Cs can diffuse through the Pb layers, even through the thicker Pb layer in the Pb/Cs sample, while halide diffusion and homogenization are hindered.

The movement of halides, particularly Br^–^, is largely governed by FA^+^, which acts as its primary source. In this context, previous studies on hybrid perovskite formation have shown that Cs^+^ effectively participates in the intercalation of FA^+^, thereby facilitating conversion to the perovskite phase.^33^ Other reports have also highlighted the beneficial role of alkali metals such as Cs^+^ and K^+^ in promoting halide homogenization.^34,35^ Consistently, CsBr-free scaffolds lead to poorly crystalline and PbI_2_-rich perovskite films, as shown in Figure S3. For the case of Pb/Cs, it seems that the separation of PbI_2_ and CsBr into two layers affects the FA^+^ diffusion and therefore halide distribution, potentially leading to localized PbI_2_-rich regions, as observed by XRD. On the other hand, we also observe that the coevaporated and Pb/Cs/Pb/Cs/Pb samples still show a moderate Br^–^ accumulation toward the center of the perovskite layer, suggesting that the Cs^+^ mixing is not the only factor playing a role in the halide homogenization.

To test the hypothesis of the limited FA^+^ diffusion, we converted the same Pb/Cs scaffold stack using FA^+^ solutions of increasing concentration. Figure S4 compares the XRD patterns of perovskites obtained from Pb/Cs stacks converted with 0.65, 0.70, and 0.75 M alcohol-based FA^+^ solutions. A step increase of just 0.05 M is sufficient to induce significant changes in the perovskite diffraction pattern. As expected, higher FA^+^ concentrations promote a more complete intercalation, resulting in progressively lower residual PbI_2_ content. Notably, when a 0.75 M solution is used, the PbI_2_ reflection disappears entirely, indicating complete conversion to the perovskite phase. These results support our earlier interpretation that limited halide uniformity, particularly in the Pb/Cs sample, stems from insufficient FA^+^ penetration. By enhancing FA^+^ availability during conversion, we effectively mitigate the localized PbI_2_-rich regions previously observed by XRD. Furthermore, the photoluminescence (PL) shift and broad full width at half-maximum (fwhm), discussed later in this work, can also be attributed to the pronounced spatial separation of halides in these samples, particularly in the Pb/Cs case. One possible strategy to mitigate this issue involves modifying both the scaffold and the solution composition. Specifically, introducing Br^–^ via PbBr_2_, while adjusting the FABr content accordingly, could promote a more uniform halide distribution.^6^

As shown in Figure 3d–f, the morphology of the perovskite film is strongly influenced by both the scaffold deposition method and the number of Pb and Cs layers. Cross-sectional and top-view scanning electron microscopy (SEM) images identify a pronounced roughness in the perovskite derived from the Pb/Cs scaffold, in contrast to the smoother surfaces and comparable grain sizes observed in films obtained from coevaporated and Pb/Cs/Pb/Cs/Pb scaffolds. The rougher surface of the Pb/Cs sample may result from the low, though non-negligible, solubility of CsBr in ethanol, as previously reported.^5^ During the spin-coating step, partial removal of the CsBr layer could occur, thereby compromising perovskite conversion and increasing surface roughness.

Furthermore, the microscopic morphology of the film influences its macroscopic appearance, as illustrated in Figure S5, which compares the pictures of devices fabricated using Pb/Cs, Pb/Cs/Pb/Cs/Pb, and Cs/Pb/Cs scaffolds. In the case of the Cs/Pb/Cs sample, despite terminating with a CsBr layer, the substrate does not exhibit the same high contrast between ITO and glass areas. Instead, it displays a hazier, yet homogeneous, surface. We attribute this difference to the lower thickness of the terminating CsBr layer, which makes it less susceptible to partial removal during the spin-coating step. Interestingly, despite these considerable variations, the cross-sectional SEM images revealed the same thickness for the converted perovskites, between 320 and 330 nm (Figure S6).

Optical characterization of the perovskite layers reveals important deviations in both the PL spectra and the optical bandgap. Analysis of the normalized PL spectra from the perovskite films on glass/ITO/SAM substrates with the six different inorganic scaffolds, shown in Figure 4a, reveals distinct trends in peak position and fwhm, which are summarized in Figure 4b. Compared to the reference perovskite, the Pb/Cs sample exhibits a notable red shift to 755 nm and a broadened fwhm, as evidenced by the presence of a shoulder peak clearly visible in Figure 4a (orange line). We attribute these features to the inhomogeneous halide distribution and incomplete PbI_2_ utilization discussed earlier.

**Figure 4:**
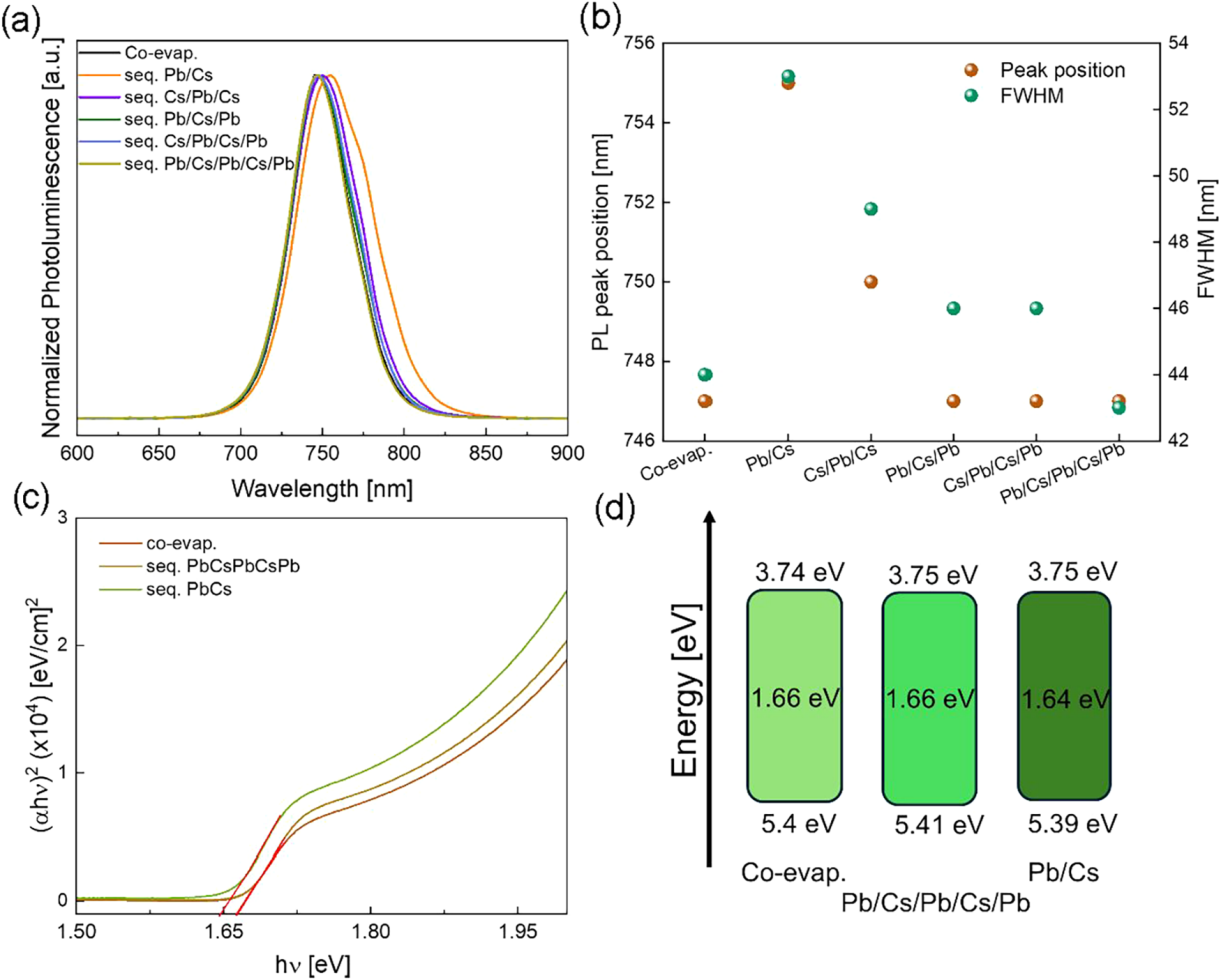
(a) Perovskite normalized photoluminescence spectra converted from the scaffold series. (b) Representation of the extrapolated PL peak position and fwhm. (c) Perovskite Tauc plot for the coevaporated, Pb/Cs and Pb/Cs/Pb/Cs/Pb scaffolds. (d) Energy diagram for the coevaporated, Pb/Cs and Pb/Cs/Pb/Cs/Pb perovskites, based on the Tauc plot and PESA measurements.

By comparison, as illustrated in Figure S7, Pb/Cs samples converted with higher-concentration solutions (0.7 and 0.75 M) exhibited the same PL peak position (755 nm) but with narrower fwhm values of 46 and 44 nm, respectively, comparable to that of the control sample. These results complement the earlier XRD and TEM analysis, indicating that while improved conversion is achieved (evidenced by the narrower fwhm and reduced PbI_2_ peak in XRD), the Pb/Cs samples may still suffer from partial washing of the terminal CsBr layer during spin-coating. This effect likely accounts for the persistent red shift from 747 to 755 nm.

By increasing the number of Pb and Cs layers in the scaffold, the PL peak progressively blue-shifts back toward 747 nm, matching the emission of the perovskite derived from the coevaporated scaffold. Concurrently, the fwhm narrows to values comparable to the control, indicating improved compositional homogenization in these multilayer films. The bandgap of each perovskite film was also calculated from Tauc plots derived from absorbance measurements, as shown in Figure 4c. While the perovskites obtained from the coevaporated and Pb/Cs/Pb/Cs/Pb scaffolds exhibit the same bandgap of 1.66 eV, the Pb/Cs-derived perovskite displays a slightly narrower bandgap of 1.64 eV.

Lastly, combining these results with photoelectron spectroscopy in air (PESA) measurements (Figure S8) allowed us to construct the band diagrams presented in Figure 4d. All samples exhibit a valence band maximum (VBM) of approximately 5.40 eV.

We integrated the five sequentially deposited scaffolds into small-area (0.1 cm^2^) solar cells using the standard p-i-n configuration illustrated in Figure 5a (see Experimental Methods). A direct device comparison fabricated from the coevaporated, Pb/Cs, and Pb/Cs/Pb/Cs/Pb scaffolds is shown in Figure 5b. All three architectures yield similar champion performances, with PCEs around 20%. The Urbach energy is extracted from the sensitive external quantum efficiency (sEQE) data measured on three randomly selected pixels, shown in Figure 5c, revealing the same value of 14 meV for each cell (Figure S9). These results indicate that despite the notable differences observed in the previous analysis, these cells have a comparable optoelectronic quality, as also confirmed by the average PCE values shown in Figure 5d.

**Figure 5:**
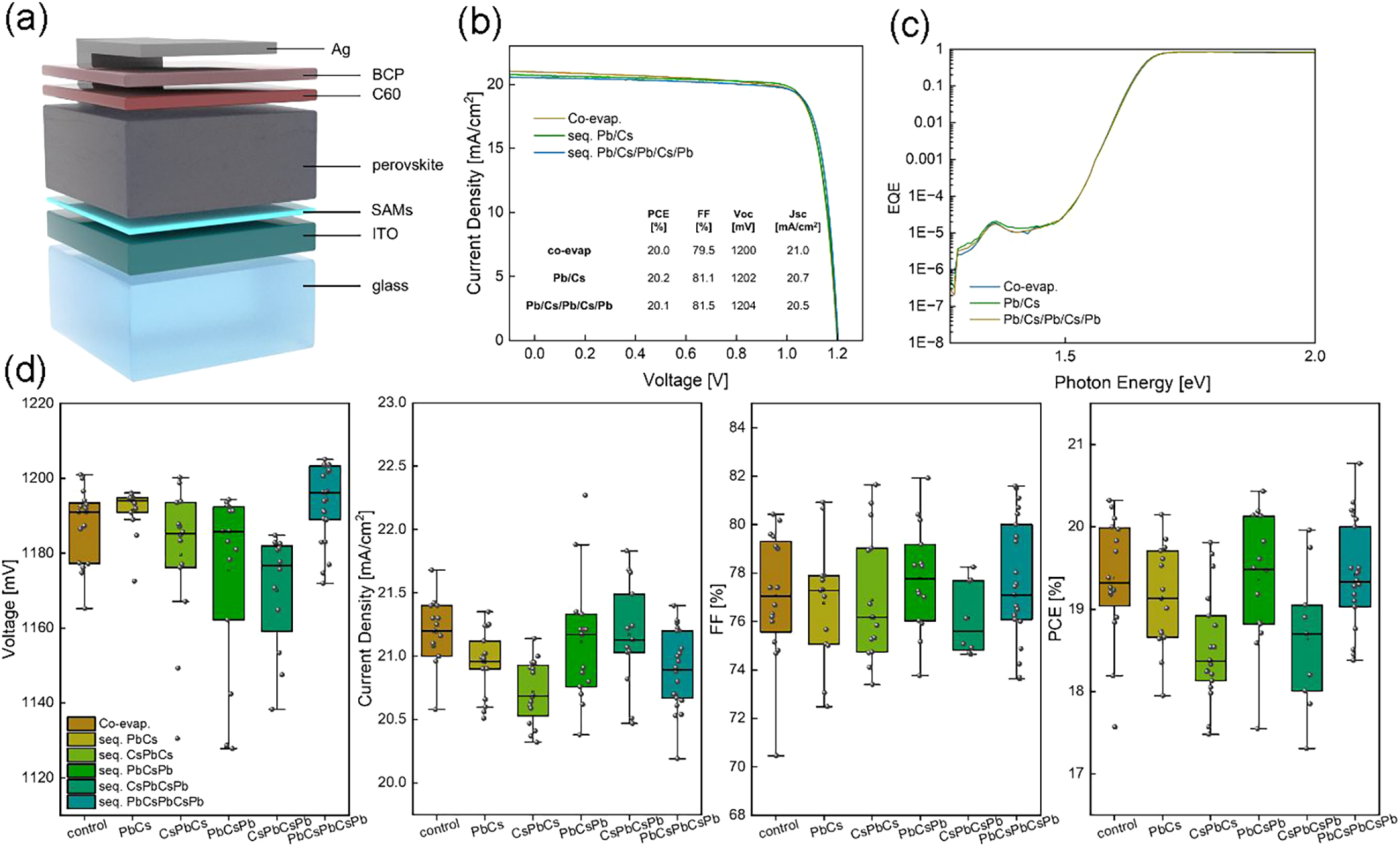
(a) p-i-n device structure used for the single junction solar cells. (b) Champion cells obtained from coevaporated, Pb/Cs, and Pb/Cs/Pb/Cs/Pb scaffolds. (c) Measured sensitive EQE from solar cells with coevaporated, Pb/Cs, and Pb/Cs/Pb/Cs/Pb scaffolds. (d) Comparison between the coevaporated and the five sequentially deposited scaffolds, when integrated into single junction solar cells.

In a wider comparison (Figure 5d), all five sequentially deposited scaffolds perform similarly to the control. Indeed, despite the large differences discussed earlier in this work, we found that all scaffolds produce an average PCE of around 19%. Nevertheless, a clear trend emerges in the open-circuit voltage (*V*_oc_). Specifically, the *V*_oc_ decreases progressively from the control down to the Cs/Pb/Cs/Pb scaffold, before recovering to nearly 1.2 V for the Pb/Cs/Pb/Cs/Pb scaffold. Multiple factors may explain the observed *V*_oc_ trend, including suboptimal perovskite conversion and halide homogenization, as well as the nature of the initial scaffold layer interfacing with the underlying SAM, where PbI_2_ may promote a more favorable buried interface after conversion. Similarly, the slightly higher *V*_oc_ observed for Pb/Cs cells could be related to the passivation effects of PbI_2_, abundant in these samples, or to the narrower bandgap, as observed earlier in this work. It is also worth mentioning that Pb/Cs scaffolds converted with higher-concentration solutions produced less efficient devices, with PCEs below 20% (Figure S10). Together, these results suggest that PbI_2_ conversion completeness, for hybrid-processed perovskites, does not necessarily translate into higher device performance. As previously mentioned in this work, the role of residual PbI_2_ remains a subject of debate; as in our experience on hybrid conversion, we have encountered both efficient and inefficient devices exhibiting significant PbI_2_ content.

Finally, the coevaporated and Pb/Cs/Pb/Cs/Pb scaffolds were used to fabricate perovskite/silicon tandem solar cells. In this configuration, the perovskite absorber layer is thicker to satisfy current-matching conditions. As a result, the coevaporation time is approximately 2.5 times longer (at the same rates) than that required for single-junction devices. In the same manner, the individual layers in the sequentially deposited scaffold are also 2.5 times thicker than their single-junction counterparts (see Experimental Section). Efficient hybrid-processed perovskite/silicon tandems commonly employ molecular additives mixed with FA^+^ salts in the alcohol solution to promote the conversion of thicker scaffolds on textured silicon. Among these additives, MACl, MASCN, 2,3,4,5,6-pentafluorobenzylphosphonic acid, and urea have been reported, leading to PCEs well above 30%.^4,36,37^ In this study, we opted not to use any additive to fairly compare the conversion properties of the coevaporated and sequential scaffolds on textured surfaces.

From the cross-section SEM images shown in Figure 6a, we observe a conformal coating of the silicon pyramids (1–1.5 μm). On the other hand, in the absence of additives such as MACl or urea, we observe an incomplete PbI_2_ reaction, localized in the silicon valleys, for the coevaporated and, to a lesser extent, for the sequentially deposited sample.

**Figure 6:**
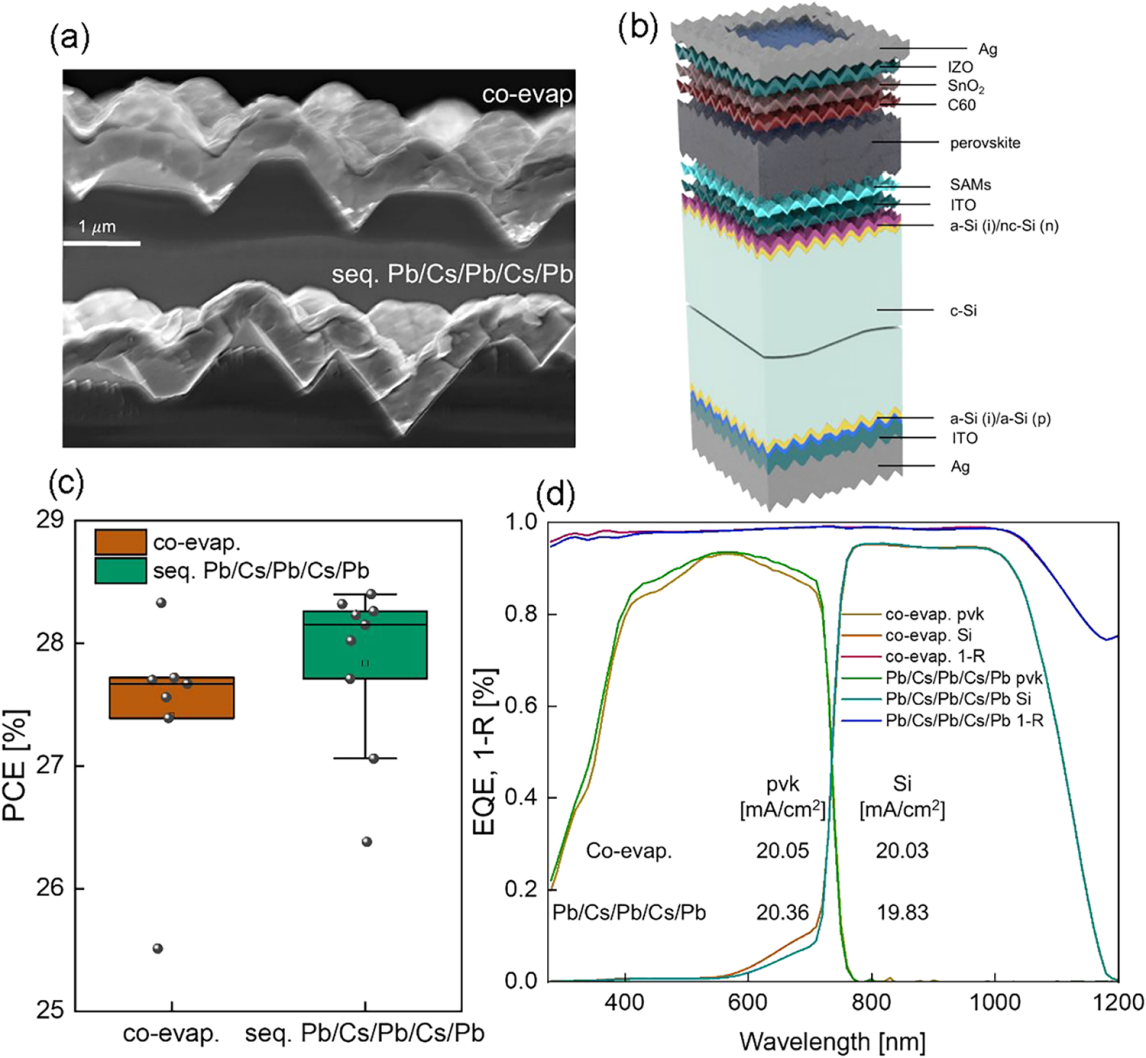
(a) Cross-sectional SEM perovskite images for the coevaporated and Pb/Cs/Pb/Cs/Pb scaffolds, highlighting the conformal coverage and remnant PbI_2_ in the silicon valleys. (b) Perovskite/silicon tandem architecture used in this work. (c) PCE comparison of the tandem cells. (d) EQE and 1-R of the tandem cells.

In this work, we employed the device architecture illustrated in Figure 6b, and as can be seen in Figure 6c, both tandems delivered similar PCE close to 28%, with a champion efficiency respectively of 28.3% for the coevaporated and 28.4% for the sequentially deposited scaffolds. However, we notice that on average, Pb/Cs/Pb/Cs/Pb scaffold delivered a higher PCE, which is to be attributed to a higher short-circuit current (*J*_sc_) and fill factor (FF) as seen in Figure S11. From the EQE shown in Figure 6d, we observe better light absorption mainly in the blue region, for the Pb/Cs/Pb/Cs/Pb sample, bringing these tandems toward a slight silicon current-limited condition, which in turn, supports a higher FF.^38^ We note here that the better EQE behaves similarly to that of the single junction devices (Figure S12), where a higher blue response is also observed. Although for single-junction devices it is difficult to establish a relationship with the average *J*_sc_ values shown in Figure 5d, due to batch-to-batch variations, a large *J*_sc_ spread, and a limited EQE data set, we hypothesize that this phenomenon might arise from lower ionic losses, as the hysteresis index (HI) illustrated in Figure S13, suggests.^39^

Due to the lack of molecular additives and highly concentrated PDAI_2_ surface passivation, the fresh tandems feature a strong HI of 0.18 and 0.13%, respectively, for the coevaporated and Pb/Cs/Pb/Cs/Pb devices, corresponding to more than 2.5% absolute PCE difference. Interestingly, we found that after periodical measurements over about 100 h of shelf storage, the HI decreases to approximately 0.08%, suggesting surface reorganization of the passivation molecules and bulk assessment of the mobile ions.^40^ At the same time, after an initial increase, both PCEs stabilize and converge around 28%. Collectively, these results further confirm the application of sequentially deposited inorganic scaffolds for hybrid-processed single junction and tandem devices, strengthening their potential for industrial translation.

## Conclusions

In this work, we explored the potential of sequentially depositing the inorganic scaffold in the vacuum–solution hybrid perovskite deposition method as a viable alternative to conventional coevaporation. Five different sequentially deposited scaffolds were compared with a coevaporated reference, revealing notable differences in PbI_2_ utilization (conversion to the perovskite phase), halide distribution, and surface morphology. Despite these variations, all sequential scaffolds exhibited similar performance in single-junction perovskite solar cells, achieving PCEs around 19%.

Based on these findings, the Pb/Cs/Pb/Cs/Pb sequential scaffold was selected for integration into perovskite/silicon tandem devices. This approach achieved a champion tandem PCE of 28.4% without molecular additives (e.g., urea, MACl), comparable to the coevaporated reference (28.3%), and with a higher average efficiency. These results demonstrate that sequential scaffold deposition offers a simpler, robust, and industry-compatible alternative to traditional coevaporation while maintaining high device performance.

## Experimental Section

### Single Junction Device Fabrication

Prepatterned glass/ITO substrates were exposed to a UV–O_3_ lamp for 15 min. Subsequently, the HTL, consisting of a mixture of Me4-PACz and MeO4-PACz in the ratio of 8:2 and a total concentration of 1 mg/mL, is spin-coated, followed by 5–7 min annealing at 100 °C and IPA dynamic washing.

For the coevaporated scaffolds, PbI_2_ and CsBr are thermally evaporated, respectively, at rates of 1 and 0.1 Å/s resulting in thin films of approximately 245 nm, of which approximately ∼220 nm are of PbI_2_ (90%) and ∼24 nm of CsBr (10%). For the sequentially deposited scaffolds, the same rates of 1 and 0.1 Å/s respectively for PbI_2_ and CsBr are used. To maintain the same stoichiometry, the thickness of each PbI_2_ and CsBr layer is adjusted according to their relative number of repetitions in the stack. For example, for the Cs/Pb/Cs scaffold, the CsBr thickness in each layer is ∼ 12 nm, while for the Pb/Cs/Pb/Cs/Pb scaffold, the thickness of each PbI_2_ layer is ∼74 nm.

The PbI_2_/CsBr scaffold is converted via spin coating using a 0.65 M solution in ethanol, of which 60% is FABr and 40% is FAI, and annealed at 150 °C for 15 min at 30% RH.

After cooling, a 0.25 mg/mL PDAI_2_ solution in IPA is spin-coated onto the substrates and annealed at 100 °C for 3–5 min. Afterward, 20 nm of C_60_ and 6 nm of BCP are thermally evaporated, followed by 120 nm of silver.

Silicon heterojunction bottom cells are fabricated on float-zone double-side-textured 4-in. wafers (n-doped; 1–5 Ω cm resistivity, 280–300 μm thickness). A random pyramidal texture was etched on both sides of the wafer, producing a pyramid size distribution ranging from 1 to 4 μm, using a KOH aqueous solution (SINGULUS SILEX). The silicon surface was then cleaned through an O_3_-based wet-chemical cleaning process. Following this, a stack of intrinsic/doped amorphous silicon passivation layers was deposited on both sides using plasma-enhanced chemical vapor deposition (PECVD, INDEOTEC OCTOPUS II), powered at 13.56 kHz, at 200 °C, with a gas mixture of hydrogen, trimethylboron, silane, and phosphine. The intrinsic layers were set to a thickness of 6 nm, while the *p*-doped and *n*-doped layers were each set to 12 nm. The back electrode was formed by sequentially sputtering ITO and Ag (120 and 250 nm, respectively) through a shadow mask with a defined aperture of 1.1 × 1.1 cm^2^. Additionally, a layer of Ag was screen-printed on top of the sputtered Ag. This was followed by a curing step in ambient air at 200 °C for 15 min. Further, the 4-in. wafers were laser-patterned (532 nm, nanosecond laser) and cleaved into 2.2 × 2.2 cm^2^ square substrates, enabling their integration into tandem solar cell architectures.

The same SAMs mixture was utilized for the tandem devices. Here, the coevaporated scaffold has a thickness of 600 nm, which is approximately 2.5 times higher than that for single junction. Similarly, the individual thicknesses of the PbI_2_ and CsBr layers in the Pb/Cs/Pb/Cs/Pb scaffold for tandem application are instead 185 and 30 nm. The concentration of the ethanol solution was adjusted to 0.72 M, and no additives were added. The same conversion step used for single junctions is applied here. A PDAI_2_ solution in IPA/CB (50/50) with a concentration of 0.75 mg/mL was used as surface passivation. Subsequently, 15 nm of C_60_ and SnO_2_ were respectively deposited via thermal evaporation and atomic layer deposition (ALD), followed by approximately 50 nm of sputtered IZO. Finally, 350 nm of Ag were thermally evaporated with a shadow mask.

### X-ray Diffraction

The XRD measurements are performed on a Bruker D2 phaser, operating in the Bragg–Brentano geometry in the 2θ range of 10–35, steps of 0.015°, dwell time of 0.200 s, and with a Cu K_a_ λ = 0.15418 nm incident radiation.

### Photoluminescence and UV–vis Measurements

The PL measurements were conducted using hyperspectral fluorescence microscopy (Photon etc. IMA) equipped with a 20× magnification microscope. The samples were placed upward and exposed to 532 nm lasers.

The light absorption of the perovskite films is performed on an Agilent Cary 5000 UV–vis-NIR spectrometer. The substrates for both PL and Abs are full-area glass/ITO/SAM.

### Transmission Electron Microscopy (TEM) and Focused Ion Beam (FIB)

For the transmission electron microscopy (TEM)-based study, a cross-sectional electron-transparent lamella was prepared using a dual-beam focused ion beam (FIB) system (Helios G4 DualBeam, FEI) equipped with an EasyLift nanomanipulator and a gallium (Ga) ion source. To protect the region of interest during FIB processing, a two-step protective coating was applied: a 0.5 μm layer of tungsten (W) deposited via electron beam (e-beam), followed by a 3 μm W layer deposited using the ion beam. The lamella was thinned to approximately 60 nm through a sequential ion beam milling process, with beam currents progressively reduced from 2.4 nA to 0.025 nA and accelerating voltages decreased from 30 kV to 5 kV to minimize ion beam-induced damage. A final low-current cleaning step (5–2 kV, 81–28 pA) was performed to remove surface contamination. TEM imaging and analysis were conducted using a double Cs aberration-corrected ThermoFisher Titan THEMIS Z 60–300 Cubed TEM microscope operated at 80 kV, with data processing performed using Gatan Digital Micrograph and Thermo Scientific Velox software suites. Perovskite samples for TEM and FIB analysis were prepared on Si/ITO/SAM substrates and covered with 100 nm of Ag.

### Photoelectron Spectroscopy in Air (PESA)

PESA was performed using a Riken Keiki AC-2 spectrometer at a UV light intensity of 50 nW. After transferring the samples to the measurement chamber, data was collected under ambient conditions. To determine the VBM levels of the perovskite/SAM/ITO samples, the 2.fifth root (CPS^0.4^) of the photoelectron yield was plotted against the incident photon energy; the emission threshold was then identified as the intersection between the baseline and the linear fit of the yield signal.

### Scanning Electron Microscopy

The perovskite samples were fabricated on full-area Si/ITO substrates. The SEM images were recorded with a Zeiss AURIGA microscope. The images were recorded with a x25 and x50 K magnification and working distance of approximately 6 mm under a 5 kV electron beam.

### Solar Cells Characterization

The solar cells with an active area of 0.1 cm^2^ were measured in a glovebox, without any preconditioning. The JV scans for each pixel were recorded in the reverse–forward sequence, between −0.1 and 1.2 V, at a scan speed of 0.2 mV/s with a Keythley 2400 and home-built software. The light intensity of the solar simulator (Abet Technologies Sun 3000 Solar Simulator) was carefully calibrated to AM1.5 with a Si reference cell. The *JV* curves of Si/perovskite tandems solar cells were measured by a Wavelabs Sinus 220 LED-based solar simulator in air without encapsulation, which was calibrated by a Fraunhofer ISE-certified calibration cell. The active area was determined by a 1.04 cm^2^ mask. The scans were carried out from −0.1 to 2.0 V with a scan rate of 150 mV/s. The cells were kept in air, indirectly exposed to indoor light for the time of measurement. A short (10–20 s) light soaking time was included before measuring the JV curves.

The sensitive EQE spectra of the single-junction perovskite solar cells were collected without external electrical or optical bias using focused monochromatic illumination from a tungsten halogen lamp combined with a monochromator (Quantum Design MSH-300) and an assembly of long-pass filters. The light was modulated by an optical chopper at 279 Hz. The device output current was amplified by a Stanford Research SR570 current preamplifier and demodulated using a Stanford Research SR830 lock-in amplifier. The lamp intensity was calibrated with a reference Si photodiode. The Urbach energy was extracted from the local derivative of the EQE data as per the following eq 1:

1 $$E_{U}={\left(\frac{d\;\ln\left(\mathrm{EQE}\right)}{dE}\right)}^{-1}$$

EQE measurements of the tandem devices were performed using pv-tools LOANA equipment, in air. When measuring perovskite top cells, the tandem devices were light-biased by infrared LED light (930 nm); when measuring silicon bottom cells, the tandem devices were light-biased by a blue LED light (440 nm) to selectively saturate the subcell and ensure accurate spectral response of the target junction. The EQE and reflectance spectra were calibrated automatically against a certified silicon reference photodiode, and the measurements were carried out under ambient conditions in a dark chamber to minimize stray light contributions.

## Supplementary Material



## Notes

Additional STEM, XRD, PL, and SEM data. Supporting device data (PDF)

## References

[1] Castriotta L. A. (2026). Entering
the 27% Era: Practical Design Rules for Single-Junction Perovskite
Solar Cells. ACS Energy Lett..

[2] Jia L., Xia S., Li J., Qin Y., Pei B., Ding L., Yin J., Du T., Fang Z., Yin Y., Liu J., Yang Y., Zhang F., Wu X., Li Q., Zhao S., Zhang H., Li Q., Jia Q., Liu C., Gu X., Liu B., Dong X., Liu J., Liu T., Gao Y., Yang M., Yin S., Ru X., Chen H., Yang B., Zheng Z., Zhou W., Dou M., Wang S., Gao S., Chen L., Qu M., Lu J., Fang L., Wang Y., Deng H., Yu J., Zhang X., Li M., Lang X., Xiao C., Hu Q., Xue C., Ning L., He Y., Li Z., Xu X., He B. (2025). Efficient Perovskite/Silicon Tandem with Asymmetric
Self-Assembly Molecule. Nature.

[3] Wu W., Gao H., Jia L., Li Y., Zhang D., Zhan H., Xu J., Li B., Geng Z., Cheng Y., Tong H., Pan Y., Liu J., He Y., Xu X., Li Z., He B., Zhou M., Wang L., Qin C. (2025). Stable and Uniform
Self-Assembled Organic Diradical Molecules for Perovskite Photovoltaics. Science.

[4] Er-raji O., Messmer C., Pradhan R. R., Fischer O., Hnapovskyi V., Kosar S., Marengo M., List M., Faisst J., Jurado J. P., Matiash O., Pasanen H. P., Prasetio A., Vishal B., Zhumagali S., Pininti A. R., Gupta Y., Baretzky C., Ugur E., Petoukhoff C. E., Bivour M., Aydin E., Azmi R., Schön J., Schindler F., Schubert M. C., Schwingenschlögl U., Laquai F., Said A. A., Borchert J., Schulze P. S. C., De Wolf S., Glunz S. W. (2025). Electron Accumulation across the
Perovskite Layer Enhances Tandem Solar Cells with Textured Silicon. Science.

[5] Liu Z., Yang S., Tian Y., Jiang L., Li G., Yao J., Liu M., Xiong Z., Tang C., Zhang H., Jen A. K.-Y., Yao K. (2026). Homogeneity Regulation in Sequential
Fabricated Perovskite Film for Industrial-Scale Deposition of Fully-Textured
Perovskite/Silicon Tandem Cells. Adv. Mater..

[6] Shi B., Liu P., Sunli Z., Han W., Sun C., Liu Y., Luo Y., Si J., Du P., Zhang F., Yang M., He Y., He B., Zhang D., Du X., Xu X., Xia R., Zhang X., Chen Y., Gao J., Zhao Y., Zhang X. (2025). Halogen Anion Pre-Homogenization of Sequentially Deposited Wide Bandgap
Perovskites for Commercial Textured Perovskite/Silicon Tandem Solar
Cells. Energy Environ. Sci..

[7] Gao Y., Wang Y., Yang P., Shi B., Liu Z., Liu S., Li S., Liu Y., Ge X., Liu P., Luo Y., Sun C., Du X., Wang P., Zhao Y., Shao J., Zhang X. (2025). Shear Flow
Strategy for Coating Homogeneity
of Organic Materials in Perovskite Solar Cells and Modules. Joule.

[8] Er-raji O., Said A. A., Subbiah A. S., Hnapovskyi V., Vishal B., Pininti A. R., Marengo M., Bivour M., Kohlstädt M., Borchert J., Schulze P. S. C., De
Wolf S., Glunz S. W. (2025). Coating Dynamics in Two-Step Hybrid Evaporated/Blade-Coated
Perovskites for Scalable Fully-Textured Perovskite/Silicon Tandem
Solar Cells. EES Solar.

[9] Siegrist S., Yang S.-C., Gilshtein E., Sun X., Tiwari A. N., Fu F. (2021). Triple-Cation Perovskite Solar Cells
Fabricated by a Hybrid PVD/Blade
Coating Process Using Green Solvents. J. Mater.
Chem. A.

[10] Pesch R., Petry J., Petermann J., Pappenberger R., Kuechle T., Schenck J., Rothbauer L. P., Fang L., Liu X., Rafizadeh S., Nejand B. A., Sutter J., Lemmer U., Paetzold U. W. (2025). Efficient
Perovskite/Silicon Tandem Solar Cells Using Hybrid Two-Step Inkjet
Printing with Edge Isolation Precision. Small
Sci..

[11] Yu T., Zhang M., Jiang Y., Zhang S., Zhou Z., Yin L., Shi L., Liang S., Dong Y. (2026). A Two-Step Hybrid Evaporation-Solution
Method for Fabricating Large-Area High-Efficiency Perovskite Solar
Cells. Org. Electron..

[12] Zheng X., Kong W., Wen J., Hong J., Luo H., Xia R., Huang Z., Luo X., Liu Z., Li H., Sun H., Wang Y., Liu C., Wu P., Gao H., Li M., Bui A. D., Mo Y., Zhang X., Yang G., Chen Y., Feng Z., Nguyen H. T., Lin R., Li L., Gao J., Tan H. (2024). Solvent Engineering for Scalable
Fabrication of Perovskite/Silicon Tandem Solar Cells in Air. Nat. Commun..

[13] Shen C., Hu Y., Zhou S., He Z., Han J., Zhang D., Lu L., Mo X., Zhang Q. (2025). Vacuum Thermal
Evaporation for OLEDs:
Fundamentals, Optimization, and Implications for Perovskite LEDs. Adv. Electron. Mater..

[14] Rezaei-Hartmann, N. ; Brand, T. ; Groß, C. ; Malguth, E. ; Mathies, F. ; Meitzner, R. ; Ronsin, O. ; Segadlo, K. ; Tarasov, A. ; Unger, E. ; Camus, C. Unveiling the Chemistry: A Dive into Wet Chemical Perovskite Thin Film Creation Revealed by Spectral in-Situ Reflectance. In 2024 IEEE 52nd Photovoltaic Specialist Conference (PVSC) 2024; pp 456–458 10.1109/PVSC57443.2024.10749151.

[15] Tian, S. I. P. ; Liu, Z. ; Chellappan, V. ; Lim, Y.-F. ; Ren, Z. ; Oviedo, F. ; Teo, B. H. ; Thapa, J. ; Dutta, R. ; MacLeod, B. P. ; Parlane, F. G. L. ; Senthilnath, J. ; Berlinguette, C. P. ; Buonassisi, T. Rapid and Accurate Thin Film Thickness Extraction via UV-Vis and Machine Learning. In 2020 47th IEEE Photovoltaic Specialists Conference (PVSC) 2020; pp 128–132 10.1109/PVSC45281.2020.9300634.

[16] Kumar R. E., Tiihonen A., Sun S., Fenning D. P., Liu Z., Buonassisi T. (2022). Opportunities for Machine Learning to Accelerate Halide-Perovskite
Commercialization and Scale-Up. Matter.

[17] Pistor P., Borchert J., Fränzel W., Csuk R., Scheer R. (2014). Monitoring
the Phase Formation of Coevaporated Lead Halide Perovskite Thin Films
by in Situ X-Ray Diffraction. J. Phys. Chem.
Lett..

[18] Held V., Mrkyvkova N., Halahovets Y., Nádaždy P., Vegso K., Vlk A., Ledinský M., Jergel M., Bernstorff S., Keckes J., Schreiber F., Siffalovic P. (2024). Evolution of Defects, Morphology, and Strain during
FAMAPbI3 Perovskite Vacuum Deposition: Insights from In Situ Photoluminescence
and X-Ray Scattering. ACS Appl. Mater. Interfaces.

[19] Mirza A., McDonald C., Svrcek V., Sai H., Fujiwara H., Murakami T. N., Matsui T. (2025). Rapid Deposition of PbI2 Precursors
in Vacuum/Solution Hybrid Process for Efficient and Industrially Feasible
Perovskite Solar Cells. Solar RRL.

[20] Petry J., Škorjanc V., Diercks A., Feeney T., Morsa A., Kimmig S. R., Baumann J., Löffler F., Auschill S., Damm J., Baumann D., Laufer F., Kurpiers J., Müller M., Korte L., Albrecht S., Roß M., Paetzold U. W., Fassl P. (2025). Industrialization of
Perovskite Solar Cell Fabrication: Strategies to Achieve High-Throughput
Vapor Deposition Processes. EES Solar.

[21] Petry J., Pappenberger R., Welle A., Zhao T., Diercks A., Pesch R., Krause M., Fassl P., Paetzold U. W. (2026). Benchmarking
Inorganic Deposition Routes for Hybrid Two-Step Processed Perovskite
Solar Cells: A Materials Perspective. Solar
RRL.

[22] Er-raji O., Mahmoud M. A. A., Fischer O., Ramadan A. J., Bogachuk D., Reinholdt A., Schmitt A., Kore B. P., Gries T. W., Musiienko A., Schultz-Wittmann O., Bivour M., Hermle M., Schubert M. C., Borchert J., Glunz S. W., Schulze P. S. C. (2024). Tailoring
Perovskite Crystallization and Interfacial Passivation in Efficient,
Fully Textured Perovskite Silicon Tandem Solar Cells. Joule.

[23] Sahli F., Werner J., Kamino B. A., Bräuninger M., Monnard R., Paviet-Salomon B., Barraud L., Ding L., Diaz Leon J. J., Sacchetto D., Cattaneo G., Despeisse M., Boccard M., Nicolay S., Jeangros Q., Niesen B., Ballif C. (2018). Fully Textured Monolithic Perovskite/Silicon Tandem
Solar Cells with 25.2% Power Conversion Efficiency. Nat. Mater..

[24] Gao Y., Raza H., Zhang Z., Chen W., Liu Z. (2023). Rethinking
the Role of Excess/Residual Lead Iodide in Perovskite Solar Cells. Adv. Funct. Mater..

[25] Chang J., Zhou Q., Wang Y., Yan W. (2026). The Residual PbI2 Paradox
in Perovskite Solar Cells: Harnessing Excess Phase for Synergistic
Performance-Stability Improvement. Small.

[26] Shi B., Yao X., Hou F., Guo S., Li Y., Wei C., Ding Y., Li Y., Zhao Y., Zhang X. (2018). Unraveling
the Passivation Process of PbI2 to Enhance the Efficiency of Planar
Perovskite Solar Cells. J. Phys. Chem. C.

[27] Chen Y., Meng Q., Xiao Y., Zhang X., Sun J., Han C. B., Gao H., Zhang Y., Lu Y., Yan H. (2019). Mechanism of PbI2 in Situ Passivated Perovskite Films for Enhancing
the Performance of Perovskite Solar Cells. ACS
Appl. Mater. Interfaces.

[28] Roose B., Dey K., Chiang Y.-H., Friend R. H., Stranks S. D. (2020). Critical Assessment
of the Use of Excess Lead Iodide in Lead Halide Perovskite Solar Cells. J. Phys. Chem. Lett..

[29] Park B.-w., Kedem N., Kulbak M., Lee D. Y., Yang W. S., Jeon N. J., Seo J., Kim G., Kim K. J., Shin T. J., Hodes G., Cahen D., Seok S. Il. (2018). Understanding
How Excess Lead Iodide Precursor Improves Halide Perovskite Solar
Cell Performance. Nat. Commun..

[30] Tumen-Ulzii G., Qin C., Klotz D., Leyden M. R., Wang P., Auffray M., Fujihara T., Matsushima T., Lee J.-W., Lee S.-J., Yang Y., Adachi C. (2020). Detrimental Effect of Unreacted PbI2
on the Long-Term Stability of Perovskite Solar Cells. Adv. Mater..

[31] Liu F., Dong Q., Wong M. K., Djurišić A. B., Ng A., Ren Z., Shen Q., Surya C., Chan W. K., Wang J., Ng A. M. C., Liao C., Li H., Shih K., Wei C., Su H., Dai J. (2016). Is Excess
PbI2 Beneficial for Perovskite Solar Cell Performance?. Adv. Energy Mater..

[32] Wang H.-Y., Hao M.-Y., Han J., Yu M., Qin Y., Zhang P., Guo Z.-X., Ai X.-C., Zhang J.-P. (2017). Adverse
Effects of Excess Residual PbI2 on Photovoltaic Performance, Charge
Separation, and Trap-State Properties in Mesoporous Structured Perovskite
Solar Cells. Chem. – Eur. J..

[33] Er-raji O., Bett A. J., Lange S., Nagel H., Bivour M., Schultz-Wittmann O., Hagendorf C., Hermle M., Borchert J., Glunz S. W., Schulze P. S. C. (2025). Toward Efficient and Industrially
Compatible Fully Textured Perovskite Silicon Tandem Solar Cells: Controlled
Process Parameters for Reliable Perovskite Formation. Prog. Photovoltaics: Res. Appl..

[34] Xiong Z., Zhang Q., Cai K., Zhou H., Song Q., Han Z., Kang S., Li Y., Jiang Q., Zhang X., You J. (2025). Homogenized Chlorine
Distribution for > 27% Power Conversion Efficiency
in Perovskite Solar Cells. Science.

[35] Correa-Baena J.-P., Luo Y., Brenner T. M., Snaider J., Sun S., Li X., Jensen M. A., Hartono N. T. P., Nienhaus L., Wieghold S., Poindexter J. R., Wang S., Meng Y. S., Wang T., Lai B., Holt M. V., Cai Z., Bawendi M. G., Huang L., Buonassisi T., Fenning D. P. (2019). Homogenized Halides and Alkali Cation
Segregation in Alloyed Organic-Inorganic Perovskites. Science.

[36] Chin X. Y., Turkay D., Steele J. A., Tabean S., Eswara S., Mensi M., Fiala P., Wolff C. M., Paracchino A., Artuk K., Jacobs D., Guesnay Q., Sahli F., Andreatta G., Boccard M., Jeangros Q., Ballif C. (2023). Interface
Passivation for 31.25%-Efficient Perovskite/Silicon Tandem Solar Cells. Science.

[37] Luo X., Luo H., Li H., Xia R., Zheng X., Huang Z., Liu Z., Gao H., Zhang X., Li S., Feng Z., Chen Y., Tan H. (2023). Efficient Perovskite/Silicon Tandem
Solar Cells on Industrially Compatible Textured Silicon. Adv. Mater..

[38] Xu L., Xu F., Liu J., Zhang X., Subbiah A. S., De Wolf S. (2023). Bandgap Optimization
for Bifacial Tandem Solar Cells. ACS Energy
Lett..

[39] Torre
Cachafeiro M., Worsley C. A., Ji F., Watson T. M., Tress W. (2026). Inverted J–V Hysteresis in Perovskite Solar Cells: Insights
from Photovoltaic Quantum Efficiency. ACS Energy
Lett..

[40] Zhang Y., Zhu Y., Hu M., Pai N., Qin T., Cheng Y.-B., Bach U., Simonov A. N., Lu J. (2022). Self-Enhancement
of
Efficiency and Self-Attenuation of Hysteretic Behavior of Perovskite
Solar Cells with Aging. J. Phys. Chem. Lett..

