# Supplementary material for: Sequential Evaporation for Scalable Hybrid Processing of Perovskite/Silicon Tandem Solar Cells
Source: ACS Appl Mater Interfaces. 2026 Jun 12;18(25):35404–14. doi: 10.1021/acsami.6c07582 (PMC13339016; doi:10.1021/acsami.6c07582)
Supplement: Supplementary file 1 [file am6c07582_si_001.pdf]

Supporting Information

Sequential Evaporation for Scalable Hybrid Processing of Perovskite/Silicon Tandem Solar Cells

Lorenzo Mardegan[1,*], Mingjie He[1], Badri Vishal[1], Thomas Allen[1], Anand Subbiah[1], Arsalan Razzaq[1], Adi Prasetio[1], Anil R. Pininti[1], Martin Bivour[2], Juliane Borchert,[2,3] Ahmed Ali Said[1], and Stefaan De Wolf[1,*].

[1]*Center for Renewable Energy and Storage Technologies (CREST), Physical Sciences and Engineering, Division (PSE), King Abdullah University of Science and Technology (KAUST), Thuwal 23955-6900, Saudi Arabia.*
[2]Fraunhofer Institute for Solar Energy Systems ISE, Heidenhofstr. 2, 79110 Freiburg, Germany
[3]Chair for Photovoltaic Energy Conversion, Department of Sustainable Systems Engineering (INATECH), University of Freiburg, 79110 Freiburg, Germany

lorenzo.mardegan@kaust.edu.sa
stefaan.dewolf@kaust.edu.sa

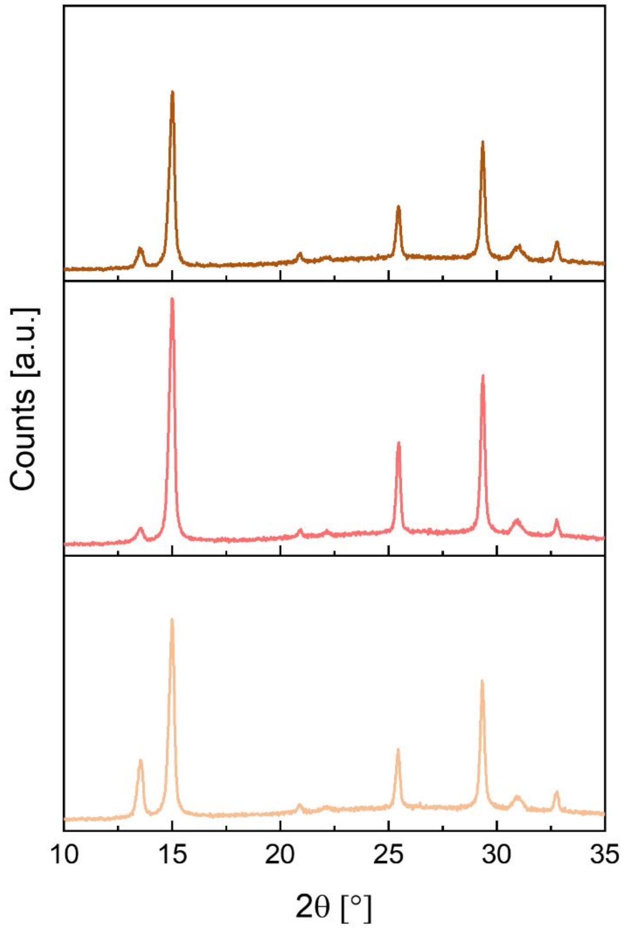

Figure S1: Perovskite XRD patterns obtained from co-evaporated scaffolds of different batches. Devices from these batches are not included in this work.

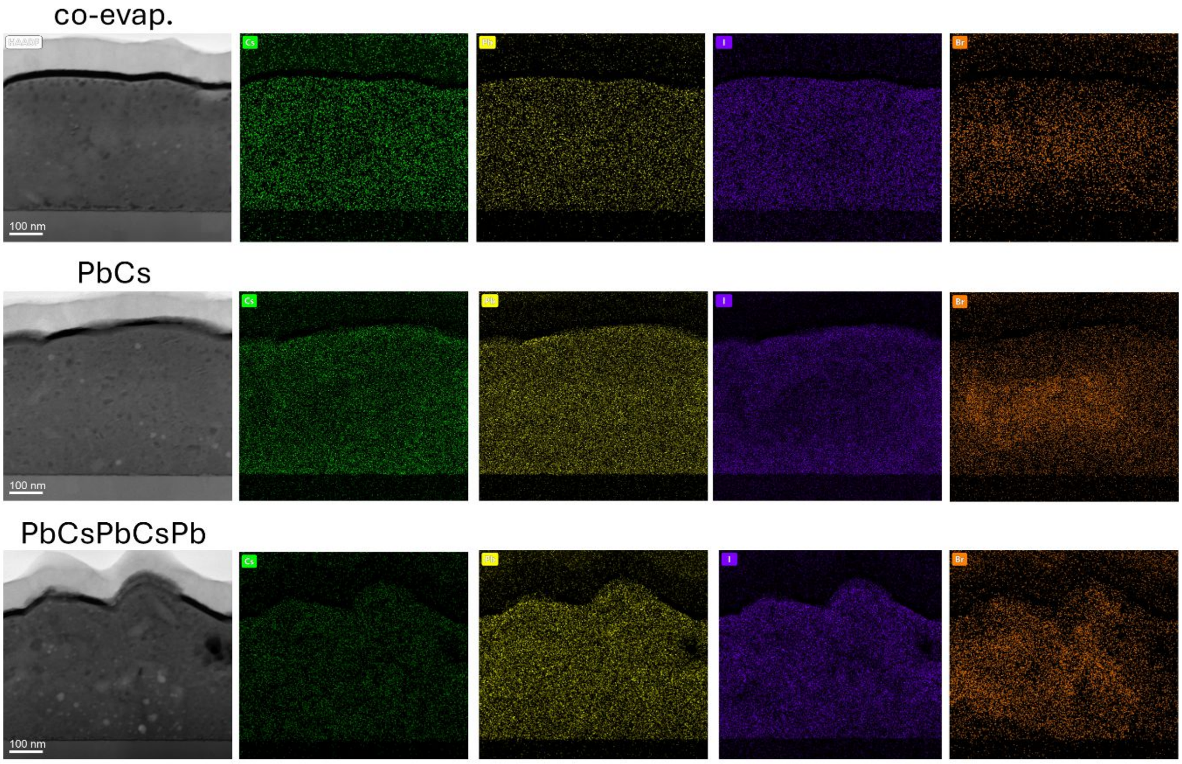

Figure S2: Zoomed-in cross sectional HAADF-STEM image of the converted perovskite on silicon substrates, with corresponding EDS elemental mapping of Cs (green), Pb (yellow), I (purple) and Br (orange).

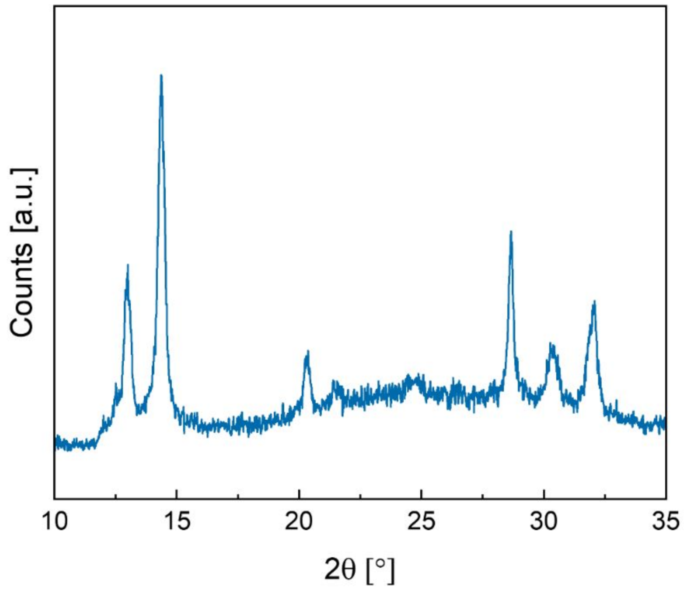

Figure S3: Perovskite XRD pattern obtained from a Pb-only scaffold

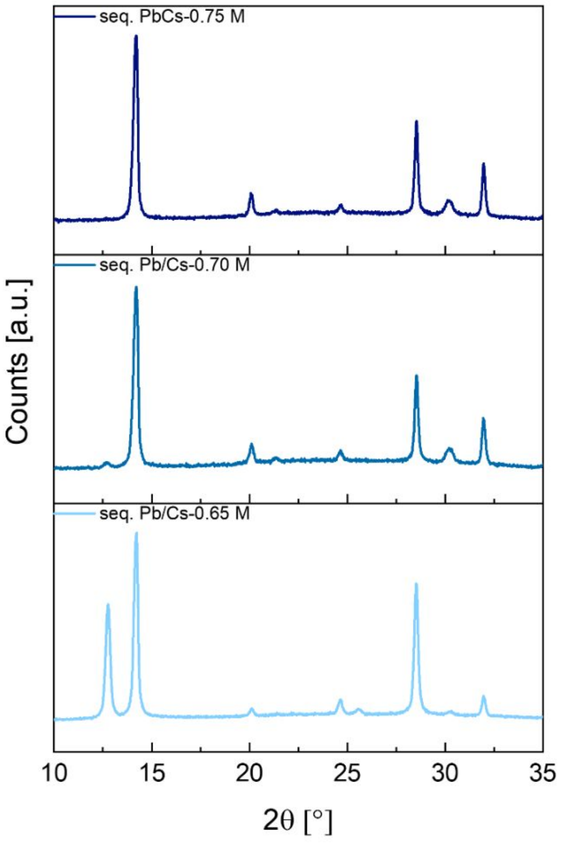

Figure S4: Perovskite XRD pattern obtained from a PbCs scaffold, converted with 0.65 M, 0.70 M, and 0.75 M FA^+ ethanol solutions.

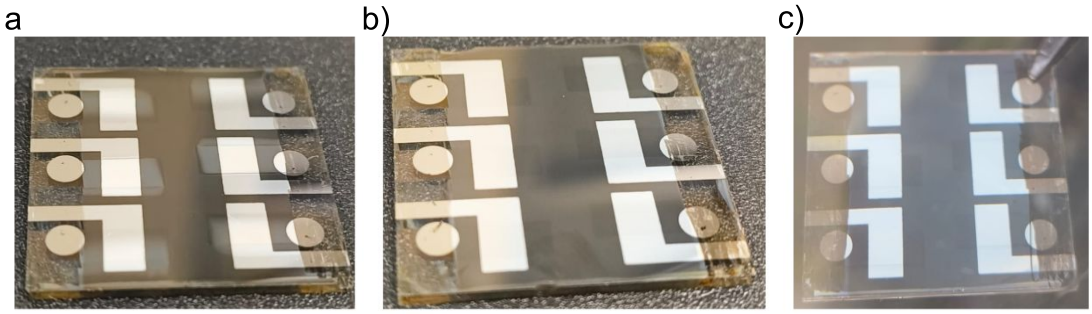

Figure S5: Photographs of complete devices obtained from a) Pb/Cs, b) from a Pb/Cs/Pb/Cs/Pb and c) Cs/Pb/Cs scaffolds, respectively.

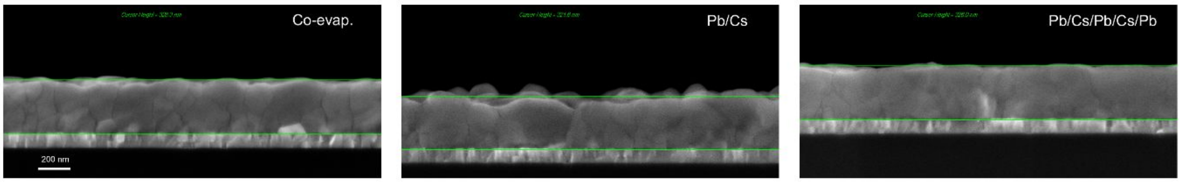

Scaffold stack	Perovskite thickness
co-evap.	328 nm
Pb/Cs	321 nm
Pb/Cs/Pb/Cs/Pb	328 nm

Figure S6: Perovskite cross-sectional SEM converted from the co-evaporated, Pb/Cs, and Pb/Cs/Pb/Cs/Pb scaffolds. Below is a table showing the measured thickness.

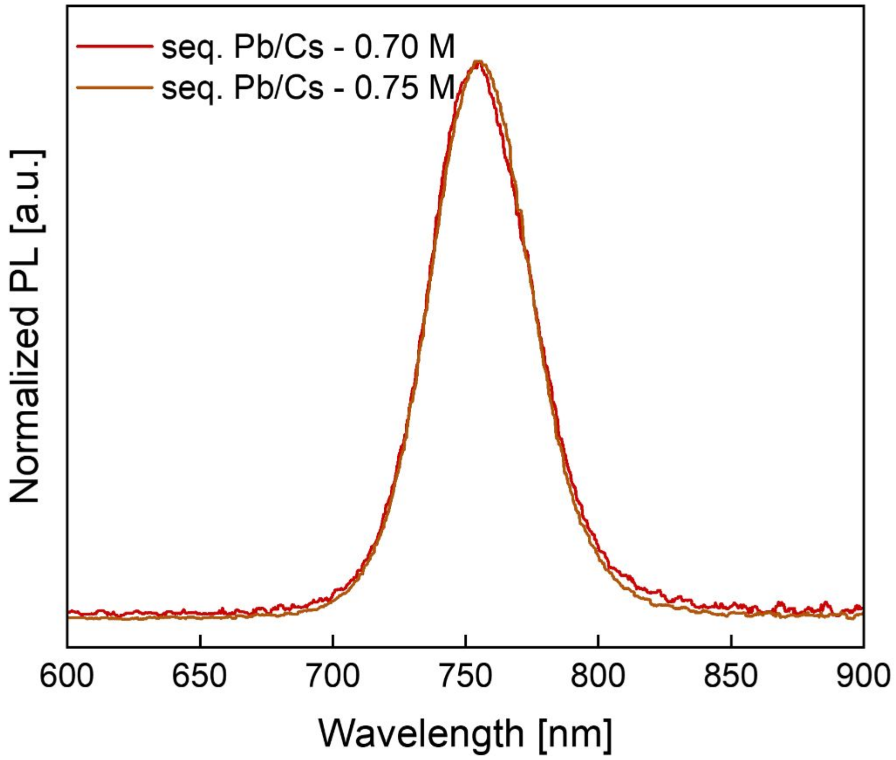

Scaffold stack	PL peak	FWHM
PbCs – 0.70 M	755 nm	46 nm
PbCs – 0.75 M	755 nm	44 nm

Figure S7: Normalized perovskite PL from Pb/Cs scaffolds converted with 0.70 and 0.75 M, with PL peak position and FWHM.

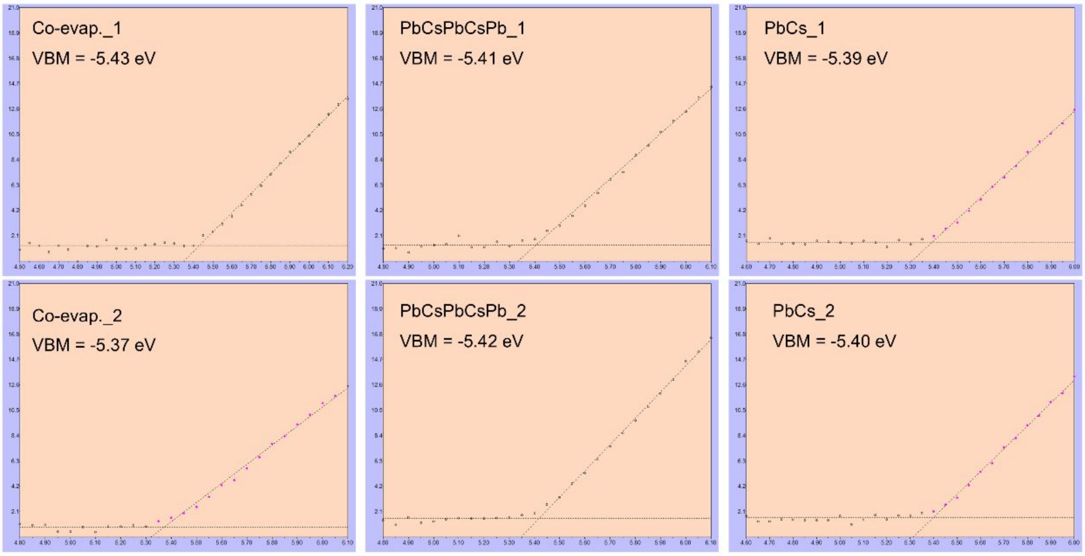

Figure S8: PESA measurements on perovskite samples converted from co-evaporated, Pb/Cs/Pb/Cs/Pb and Pb/Cs scaffolds.

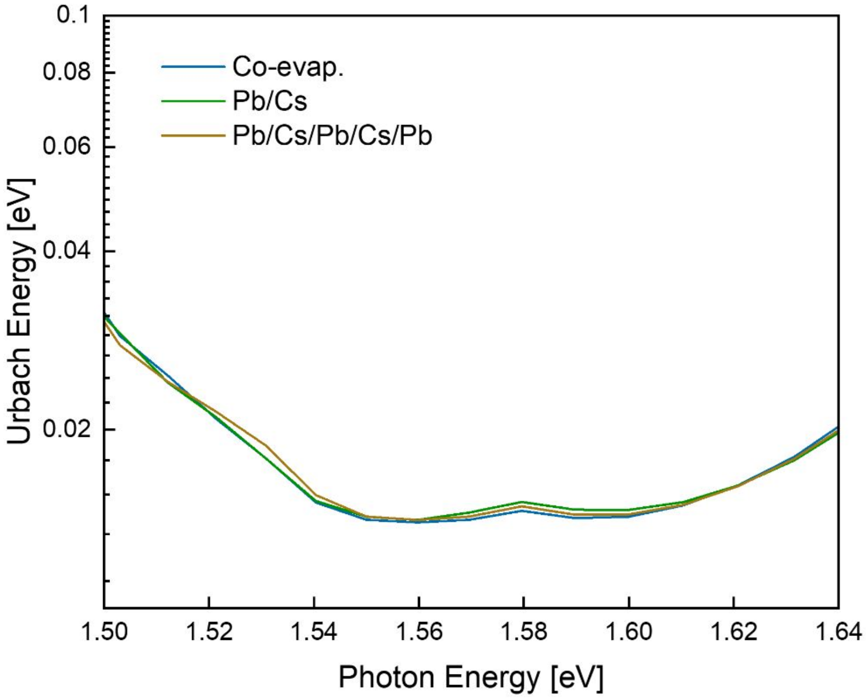

Figure S9: Urbach energy extracted from the EQE data shown in Figure 5c and S9. The calculated values are 14 meV for all samples, taken from the minima of the curve.

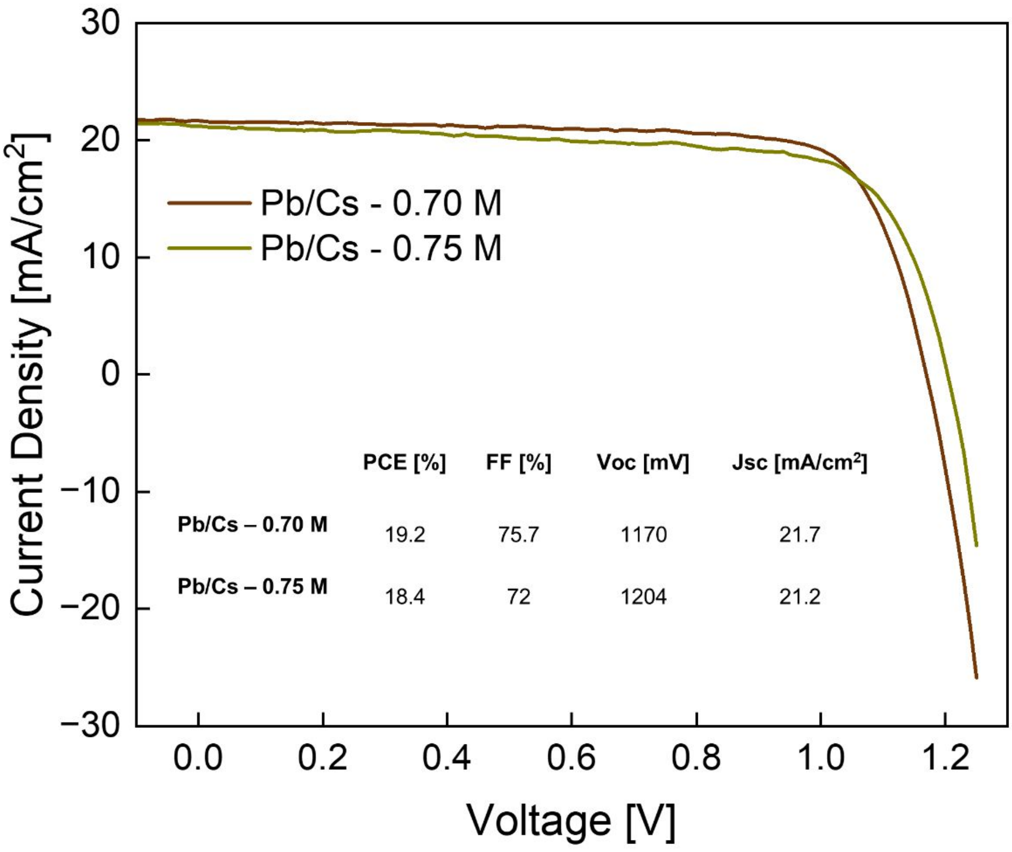

Figure S10: Best device performances for PbCs scaffolds converted with 0.70 M and 0.75 M ethanol solutions.

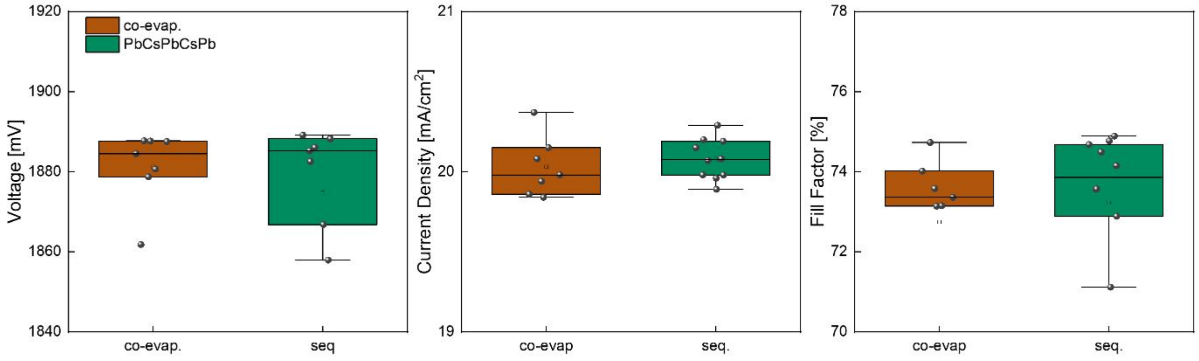

Figure S11: Measured Voc, Jsc and FF for the fresh perovskite/silicon tandems obtained with co-evaporated and Pb/Cs/Pb/Cs/Pb scaffolds.

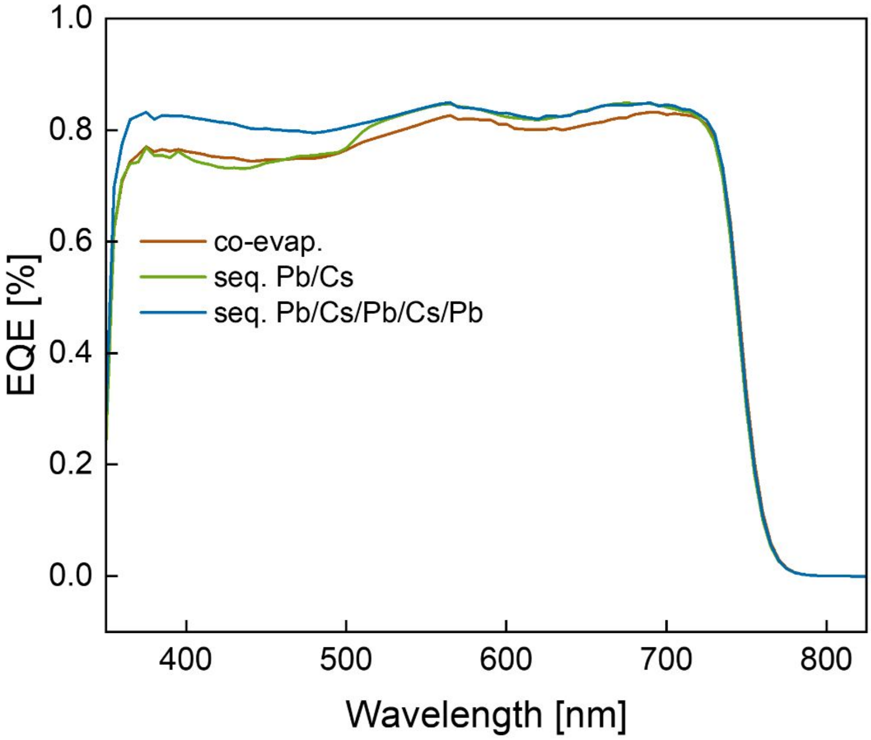

Figure S12: EQE data for Co-evap., Pb/Cs and Pb/Cs/Pb/Cs/Pb single junction devices. The EQE was measured on three randomly selected pixels.

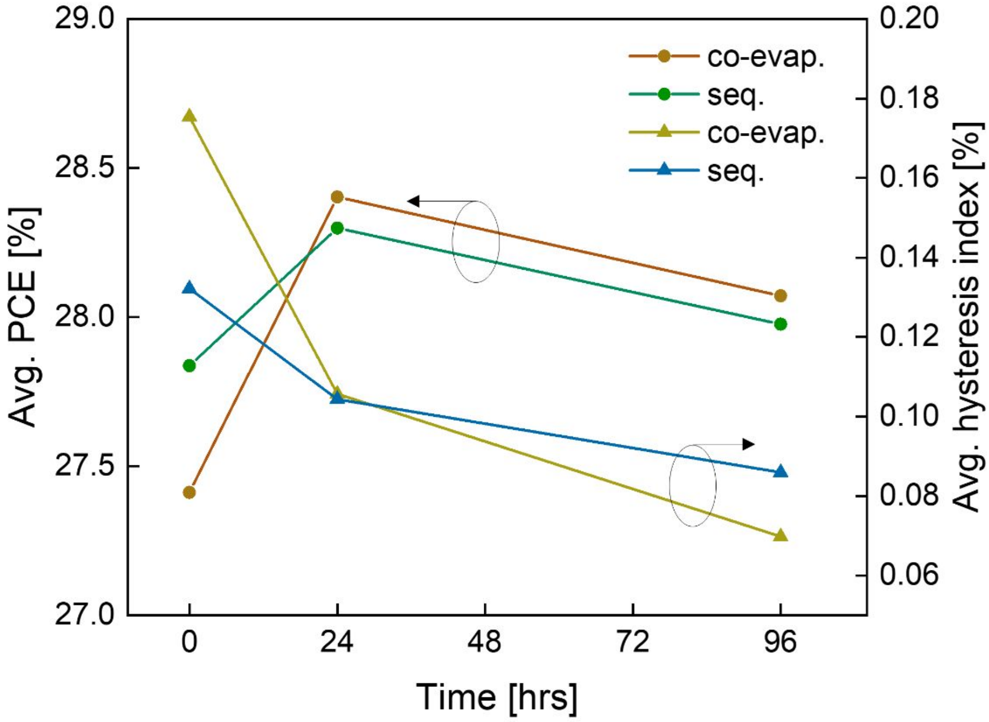

Figure S13: Tandems average PCE and HI, measured over 96 hours of dark storage.
